# Surgical Cases

**Published:** 1844-09

**Authors:** William M. Boling

**Affiliations:** Montgomery, Alabama


Art. II.—*Surgical Cases.* By William M. Boling, M.D., of Montgomery, Alabama.

*Cases of Injury of the Head.*—In injuries of the head, the small degree of external violence necessary to produce death is frequently the cause of astonishment, to persons unconnected with the medical profession—while the trifling inconvenience at times produced by the most extensive injuries of the cranium, implicating the brain itself, to a considerable extent, is no less a source of surprise to the physician.

The two first, of the following five, are cases in point:

Case I.—Nov. 14th, 1842. I was requested by Dr. Whetstone, of Autauga county, to meet him at the house of his father, (who lived some fifteen miles from town), for the purpose of operating on a negro boy, 8 years old, who had that morning received an injury of the head. The accident happened to him while driving the horses attached to the cotton gin, and was caused by his head being caught between two heavy pieces of timber, forming part of the machinery.

The accident happened early in the morning, and when I saw him, about 9 o'clock at night, he was perfectly rational—nor had he been otherwise since the occurrence. He was complaining loudly, however, of the pain. The principal injury was on the right side of the head, commencing near the centre of the coronal suture, and extending obliquely backwards and outwards across the parietal protuberance—being something over three inches in length, by two inches in width—the fragments of bone depressed more than an inch, and firmly wedged in. The inner table was fractured all round, and depressed to a greater extent than the outer table; consequently the shelving portion of the latter would prevent the insertion of an instrument under the depressed fragments. A portion of the cranium, nearly as large as a man's hand, in the centre of which lay the injured part, was completely stripped of integuments, the latter thrown back and hanging by a slip about an inch wide. On the opposite side of the head, the integuments were very much bruised and lacerated, and the cranium bared in places, but not fractured; considerable hæmorrhage had taken place.

The overshelving portions of the outer table were removed with a Heys' saw, so as to admit the introduction of an elevator under the depressed portions, and sixteen fragments of bone removed. One large piece had lacerated the dura mater, and had penetrated deeply into the cerebral substance, about a drachm and a half of which had followed its removal. The lacerated integuments were replaced and confined by a few interrupted sutures, and lint, spread with simple cerate, applied over it.

During the operation he struggled and cried lustily; and after the principal part of the fragments were removed, deep inspirations were accompanied by a collapsed and sunken state of the dura mater, while the expirations were attended by so tense a condition of the same as to cause considerable anxiety, for fear that it would burst and allow of extensive protrusion of the brain.

After the application of the dressing he was put to bed, where he slept calmly and comfortably all night.

His recovery was rapid. On the second day he was found sitting up in bed; and on the third he got up unobserved and walked out. From this time he had to be carefully watched to keep him in the house. In three weeks the scalp had almost entirely healed, and he was allowed to play about the house at his pleasure. He had scarcely any fever, and the only medicine he took was one dose of castor oil the day after the injury was received. Neither before nor after the operation was there any paralysis.

Case II.—About 4 o'clock in the afternoon, March 10th, 1841, B. B., a lad about 13 years of age, received a blow from a stone thrown by a boy something younger than himself, with whom he had quarreled about a game at marbles. The stone struck the right side of the head near the *anterior inferior angle* of the parietal bone—making a wound in the integuments, to the bone, about half an inch long, but without producing any fracture, so far as the point could be ascertained by examination with a probe.

The immediate effect of the blow was to knock him so far down, that he fell upon his hands and knees, from which, however, he immediately recovered. He was brought to my office to have the wound dressed. I merely applied a strip of adhesive plaster, and sent him home. When brought to my office, his extremities were cool, and his pulse small and weak, and about 80. He was perfectly sensible and stood up part of the time he remained. Indeed he walked part of the way home. Though a lad of a bold and daring character generally, he appeared at this time much cowed and subdued.

In about three hours after the dressing, I was requested to visit him. I found him in a stupor, from which nothing could rouse him, or cause him to evince the least consciousness, or sensibility—breathing stertorously; pulse 60, and rather weak; extremities cool; pupil of right eye very much dilated; that of the left rather contracted; paralysis of both left extremities.

He made occasional convulsive efforts to vomit, part of the matter passing through the nostrils, and part through the mouth; but the greater portion of it apparently *falling* back the œsophagus into the stomach. I was informed that after his return home he sat up some time, conversing rationally with the family. After a while some degree of drowsiness occurred, which caused him to go to bed; and in a short time he was in the situation above described.

I applied sinapisms to the epigastrium and extremities, but allowed them to remain on only a few minutes. In a couple of hours considerable reaction had taken place. His extremities were warm and his pulse about 80, full and hard, but irregular. I bled him in a full stream to 12 oz., under which his pulse softened considerably, but in the course of an hour regained its former force. Under the loss of 6oz. more of blood now, his pulse improved very much, becoming quite soft and regular. His respiration was also improved, the stupor being, for a little while, entirely removed. I took advantage of the present appearance of improvement to administer a cathartic, and mixing gr. xx of jalap and gr. x of calomel in a spoonful of thin syrup, poured it into the back part of his mouth. The larger portion of it ran passively, rather than by any effort of deglutition, down the œsophagus. In the course of the night I administered two enemata, each of which was followed by a tolerably large evacuation, both of which, together with his urine, were passed unconsciously. In less than half an hour after the second bleeding, his respiration again became stertorous, and so continued all night.

11th.—He is much as I left him last night. Pupil of right eye still very much dilated; left extremities paralysed; but he moves the right; breathing stertorous; cannot by any means be roused; skin natural, except about the head, where the temperature is a little above the healthy standard; pulse 80, soft and moderately full.

Looking upon the case as most probably one of compression caused by blood, extravasated from the middle artery of the dura mater, which I supposed to have been ruptured by the blow; and seeing no improvement produced by the depletion carried as far as the state of the pulse, &c., would admit, I proposed to trephine, in hope of being able to remove the extravasation. Before proceeding with the operation, it was proposed by his family to have a consultation. Accordingly two gentlemen of experience and high standing were called in. During the examination, the patient made an effort, in which he partially succeeded, to open the left eye. This effort was induced by a loud noise being made close to his ear, and cold water at the same time thrown in his face.

As this was the only indication of consciousness that had been elicited from the patient since early the previous evening, it was looked upon as a favorable indication, and hopes were entertained that it was the forerunner of a better state of things. The operation was postponed, and a meeting agreed upon for the next morning. During the day, as he remained in much the same condition, little was done for him, except the application of cold lotions to his head.

At 9 o'clock at night, he had a copious evacuation from his bowels, produced by an enema; after this his pulse fell to 70, and became small and weak. It soon, however, began to increase in frequency, and by 10 o'clock on the morning of the 12th, it had risen to 140, still remaining small and weak. His other symptoms remained the same. This morning a proposition to trephine was overruled. Four ounces of blood, under the loss of which his pulse became still weaker, were abstrated from his arm, and the cold applications continued to his head. In the afternoon he had two convulsive paroxysms; the convulsive action, from what I could learn, being confined principally to the muscles of the back of the neck and throat.

He continued to grow worse, and died early on the morning of the 13th.

Case III.—Dec. 23, 1840, I was requested to go to the distance of some twenty miles, in an adjoining county, to visit, in consultation, a little boy three years old, who had the evening before received a severe injury of the head, by a kick from a mule. The injury was inflicted on the right side of the frontal bone—a portion of which, of the shape of the rounded anterior extremity of the animal's foot, two and a half inches long, and more than an inch wide in the centre, was fractured, and the rounded edge depressed nearly an inch. The part opposite the depressed edge was still adherent, having been partly bent so as to admit of the depression. The physician in attendance (Dr. Turner) informed me, on my arrival, that about half an ounce of cerebral matter had escaped from the wound.

Before my arrival the depressed bone had, by the motion of the brain against it, nearly regained its natural level. The child was able to walk immediately after the accident; and as he was perfectly sensible—there being present, likewise, neither symptoms of concussion nor compression—we did not think it proper to interfere with the bone. For some inflammatory action, which supervened on the second day, and increased on the third and fourth, an active antiphlogistic course was adopted. The patient improved rapidly after the sixth day, and in a short time the wound was more a source of inconvenience than of suffering. After the complete cicatrization of the wound, a depression about the eighth of an inch remained.

Case IV.—Oct. 19th, 1842, I was requested by Dr. Pierce to visit, with him, Ben. Narrimore, a boy 14 years old, who, about two hours before, had been thrown from a horse, and had received a fracture of the cranium by a blow from the front, sharp edge of the horse's shoe, after the fall. The injury was across the middle of the right parietal protuberance, the depressed portions of bone being about two inches long, by about an inch and a quarter wide in the middle, tapering off, narrower at each end. The deepest part of the depression was about three-fourths of an inch. He got up immediately after the fall and walked home—a distance of about a quarter of a mile.

On my arrival I found him complaining very much of pain in the situation of the wound, and of a most violent headache. He was bled freely and purged, during the afternoon and night, and water dressing was applied to the wound—himself and friends positively refusing to have any effort made to remove the depressed bones.

20th.—Almost entire loss of sensation and motion of left arm, and, to a less extent, also of left leg; pupil of right eye dilated; pulse increased in frequency, and moderately full and hard. He was again bled, and took a mercurial cathartic, followed by some saline medicine; he still complained of excessive pain in the head. In the evening he remained much the same, with the exception of some spasmodic twitching of the muscles of the left arm, and of the left side of the face.

21st.—From the effect of bleeding and purging, his pulse, though frequent, is small and weak. The twitching of the muscles, noticed yesterday, has gradually increased, and has also extended to the left leg. He has now severe convulsions, confined mostly to the left side, (and more to the muscles of the left side of the face and left arm than to the leg) accompanied with great difficulty of breathing, and foaming at the mouth, but not with loss of consciousness. During the paroxysms, (some of which have lasted an hour) his countenance expresses great anxiety, but no want of intelligence. He makes efforts to speak, (in which he succeeds imperfectly) for the purpose of giving directions for the attendants to apply camphor, &c., to his nostrils, and for them to wipe away the foam from his mouth. Sometimes, as if fretted by their not understanding, or not immediately obeying his orders, he will grasp a handkerchief in his right hand and attempt to wipe the froth away from his mouth himself. After the paroxysms he appears weak, and much exhausted. His friends now, though given to understand that the probability of the operation proving beneficial is less than when first proposed, are anxious for its performance. The patient himself gives a reluctant consent, threatening me severely, with his fist and knife, if I hurt him much during the operation.

With a great deal of difficulty, the depressed fragments being small and pressed down in such a way as to support each other, like the stones of an arched well, after having removed with a small trephine a portion of sound bone, the pieces were removed by the aid of an elevator. Several large spiculæ, belonging to the inner table—extending considerably beyond the depression in the external table—loosened from the dura mater, but still slightly adherent by one of their extremities—were removed by the dressing forceps. One small spicula had penetrated the dura mater. Two or three interrupted sutures, strips of adhesive plaster, and lint, saturated with cold water, constituted the dressing.

During the operation he struggled, threatened, and fought, and called vociferously for his knife, with which to make us desist.

In an hour after the operation he had quite a severe fit, followed during the night by several others less violent. Active depletion having been carried as far as seemed safe, at the suggestion of Dr. Pierce he was put upon the use of calomel, in small doses, frequently repeated, combined with occasional doses of morphine.

At first the morphine seemed to be of service, as the spasms were for a while less violent, and occurred at longer intervals; but at length it appeared to lose its influence, though given in increased doses, (about a third of a grain every 4th hour), and was accordingly discontinued on the evening of the 23d; the patient that morning having had a paroxysm of three hours' duration. The calomel was continued in doses sufficient to keep up a moderate action on the bowels. By the evening of the 25th he was slightly ptyalized; and from that time the spasms ceased. His improvement was rapid; partial paralysis of the left arm, however, continuing for several weeks.

Case V.—T. Murrell, a lad 13 years old, of a sallow, bloated and anæmic appearance, on the 6th February, 1844, was thrown from a horse and dragged about three hundred yards, by his foot hanging in the stirrup—the horse running at full speed all the time.

When he was picked up, after his foot had been freed from the stirrup, he was quite insensible, and, according to the statement of the bystanders, entirely pulseless. When I arrived, about three-quarters of an hour after the accident, I found him lying on his side, with his body bent forward, and his thigh bent forward on the pelvis—uttering scream after scream, and writhing as if in excessive pain, (apparently abdominal) and taking no notice whatever of any question addressed him. He was badly bruised on several parts of his body, especially about the right side and shoulder, where, it was said, the horse had kicked him.

Several of his teeth were knocked out and broken off; his pulse was small and weak; extremities cool; pupils dilated, and mouth drawn rigidly to the left side. Both eyes were drawn to the left, as if from contraction of the external rectus of the left eye, and of the internal rectus of the right eye. The only appearance of injury about the head was a slight contusion on the forehead, a little to the left of the medial line, and above the superciliary ridge. Little was done for him during the night except the administration of an anodyne, as his pulse remainrd depressed, and the screaming continued.

7th.—Surface a little above the natural temperature. He remains quite stupid and continues to scream—but less frequently. Pulse weak and small; 80 in the minute. Does not lie as he did last night, with his body and thighs bent on the pelvis. ℞. ol. ricini ℥ss.

Evening.—There is as much dilatation of the pupil as I have ever seen produced by the application of belladonna. Pulse 120; rather tense; right arm cold as high as the elbow; the rest of the body hot; no evacuation; repeat ol. ricini ℥ss., and to lose ten ounces of blood; cold applications to head.

8th.—Screams less, and has sufficient sense to open his eyes and protrude his tongue when requested to do so; pupils still dilated; mouth distorted; and right arm cold, though it has been enveloped in flannel, and artificial heat applied; has had one large evacuation; repeat ol. ricini ℥ss.

9th.—Tongue dry; pulse 64, and soft; right leg as well as arm now cold; moves both slightly when pinched; but otherwise scarcely moves them at all. In other respects he remains the same; ℞. calomel gr. xx. div. in chart. vi.; one to be given night and morning.

10th.—Pulse 70, and soft; has spoken a few words rationally, this morning; continues same.

11th.—He had several operations last night, which appeared to cause him a good deal of pain; since the operations he has been quite easy; continue calomel, &c.

12th.—Gums a little sore; pulse 76; countenance improved; pupils less dilated; ate a cracker this morning; recognizes me, but not his mother.

He continued to improve slowly. By the 10th of March he was able to go about; but his gait was awkward on account of weakness in his right leg; nor has he yet entire control over the right arm. His father thinks that his intellect is slightly impaired.

*Case of Ununited Fracture.*—On the 16th of November, 1841, I was called to see a very stout, robust, and muscular stage-driver, who, in driving the stage out of town at night, had upset it, suffering in consequence a fracture of one of his clavicles, and an oblique, slightly comminuted fracture of the left femur, below the middle. The limb was dressed with a roller bandage and paste-board splints, and placed in the modification of the double inclined plane, described by Dr. Nott, of Mobile, in the November number, for 1838, of the American Journal of Medical Sciences. At the end of seven weeks the limb was removed from the apparatus. The fractured part was surrounded by a large mass of provisional callus, and appeared firm. For several days he made some awkward attempts to walk with crutches, assisted by his companions; one of whom generally supported him on each side.

Dissatisfied with the slow progress he made in *relearning* to walk, and attributing it in a great measure to want of confidence, his companions one day led him into the middle of the room, out of the reach of anything by which he might steady himself, and there left him to support himself with his crutches as best he could. He tottered for a few seconds and fell.

On examining the situation of the fracture, the ends of the bone were found quite movable, though the provisional callus appeared to have sustained no injury from the fall, the motion taking place, within the callus, between the ends of the bone, as in the capsular ligament of a joint. No grating or crepitus could be heard, as in a recent fracture, by rubbing the ends of the bone together.

The apparatus was re-applied. Upon examination at the end of five weeks, the parts were found in *statu quo.* During the whole treatment of the case he was very restless, and impatient of confinement, frequently moving and turning himself in bed; and when his companions refused to assist him, he would call in such persons as he could see passing his door, and beg of them to move him. He frequently unbuckled the straps which confined the limb to the apparatus.

By this time confinement and low diet had reduced him considerably. I put him upon a generous diet and allowed him a moderate quantity of brandy and porter.

After rubbing the ends of the bone together, till considerable pain was produced by it, I again applied the apparatus. At the end of seven weeks more I examined and found the parts apparently still more movable. The provisional callus had diminished, and appeared to partake much more of the nature of fibro-cartilage than of bone.

The poor fellow's patience and hopes seemed now completely exhausted; and, with tears in his eyes, he begged me to amputate the limb.

I now determined to try the effect of a starch bandage, if possible so arranged that he might walk about. With this intention I covered the limb from the toes up to the top of the thigh, with a simple roller applied with moderate tightness. Over this to the same extent I applied two folds of the bandage, well saturated with starch. Next I applied four splints of book-binders paste-board—well softened—reaching from the knee to the upper part of the thigh; and over these again, five or six folds of the starched bandage.

The limb was now kept in an easy and straight position for some four or five days; at the end of which time the bandage had become perfectly dry, very firm, and fitted so accurately to the limb that it was impossible for the fractuted parts to yield in any manner.

His companions now undertook the task again of teaching him to walk—and with such success this time, that, in little more than a week, he was able to get about pretty well with the assistance of a cane only. In two or three weeks more he was in the stables attending to the stage horses. His gait was vigorous, and with the exception of the stiffness of the knee—necessarily occasioned by the bandage, for the time—no palpable defect could be observed in his walk.

In four or five weeks from the time of its application, that part of the bandage covering the foot and ankle had become displaced, and the parts consequently swollen and chafed. On this account I removed the whole of the bandage and applied another in the same way. While the bandage was off I did not attempt to see whether the limb would bend or not. The last bandage remained on about six weeks. On its removal the fracture was found firmly united. During all this time he was going about working in the stables, and at no time, while doing so, did the fractured part cause him more than a very moderate degree of pain. The fractured limb was nearly half an inch shorter than the other; but the shortening had occurred before the application of the starched bandage.

*Reminiscences—and Excerpts from my note-book.*

Dec. 9th, 1841—I was called on to visit an old gentleman residing some fifteen miles from town, at whose residence I arrived late at night. After having examined his case, and prescribed, I was requested by his overseer, who lived but a short distance off, to see and prescribe for his wife.

On inquiry I found that she had been in labor over 48 hours; that for the last three or four hours the pains had gradually become less strong, with longer intervals between them, until at length they had almost entirely subsided. The presentation was natural; the head lodged in the pelvis just within the labiæ. An old negro woman, who had been officiating as midwife, informed me that the head had not advanced any during the last five or six hours. I was informed by the husband that a neighboring physician had been sent for, and was expected in half an hour—but was earnestly requested by him, in the meantime, to *do something* for her.

As I was fatigued, and wished to retire—and another physician being expected so soon to take charge of the case—I would have declined making any prescription but for the urgent solicitation of the patient and her friends. To satisfy them until the physician's arrival, I, with some gravity, administered about half a teaspoonful of spts. lav. comp., in a small quantity of water. To my inquiries, next morning, respecting the progress of the case, her husband informed me that I had scarcely left her room before the pains became strong and frequent, and that in less than half an hour—and before the arrival of the other physician—the labor had terminated favorably. What a strong case this would have been in favor of ergot, had I administered it! Or, what a triumphant case for Homœopathy, if an amateur of the science had administered a "globule!"

In the afternoon of August 26th, 1843, I visited a negro woman, who had that morning been delivered of a dead child at the seventh month. For a month before parturition she had flooded a good deal, and since delivery the hæmorrhage had had been quite alarming. I found her free from pain, (which had ceased soon after the delivery of the child) and with the uterus quite relaxed. On examination per vaginam, I found a portion of the placenta presenting at the mouth of the womb, and by gentle traction discovered that it was still adherent to the anterior part of the uterus, near the neck. After making some ineffectual efforts to induce uterine contractions, I was compelled to leave her, to attend other pressing engagements; previously, however, having drawn a portion of the detached edge of the placenta through the os uteri, and left it acting the part of a tampon. Among other things I prescribed ergot, (to be commenced with immediately) and some powders, composed of sugar of lead and opium, to be given in case the flooding should again become profuse without *a return of pain.*

Her master misunderstood my directions, and supposed that the powders were to excite uterine pain and contractions in case the ergot failed to do so. Accordingly having administered the ergot without effect—in about four hours after she had taken the last of it—he commenced giving the powders. In an hour after the administration of the first one, she commenced having pretty severe pains attended with some flooding. He continued giving them, and on visiting her the next day, I found the uterus contracted, and the placenta lying in the vagina. Her master thought the tea (infusion of ergot) worthless, but was much pleased with the powders.

In October, 1840, I attended a little girl about four years old, for a severe attack of enteritis. One of the prominent features in the case was obstinate constipation of the bowels, attended with deep-seated and constant pain in the lower part of the abdomen. Every practicing physician knows how important the free evacuation of the bowels is considered by the friends of the sick, and with what anxiety they look forward to its accomplishment, especially when their attention becomes directed to the matter by the occasional administration of an enema. Up to the 6th day of treatment, nothing like a free discharge had been procured from the bowels, notwithstanding the administration of laxatives and enemata, and once, the introduction of a large gum elastic catheter into the sigmoid flexure of the colon.

The feverish anxiety of the friends had by this time gradually mounted to a high state af excitement. A consultation was accordingly proposed, and a physician of more years and experience than myself appointed to meet me at 4 o'clock in the afternoon.

My morning prescription was two grains of calomel and half a grain of extract of hyosciamus, to be repeated every third hour—and occasionally the tepid bath. My senior anticipated the time of meeting about half an hour, and forgetting (as *old doctors* sometimes will) that some *little* courtesy was due to a junior member of the profession, had ordered a discontinuance of my prescription, and had substituted in its stead *syrup. rhei. arom.* ℥ss., *magnes. calc.* grs. iii., to be given every second hour. The first dose had been given just before my arrival. We sat beside the patient, conversing for about half an hour, when lo! she had a copious discharge from her bowels, and this was followed in the course of an hour or two by another—and another—to the perfect satisfaction of her anxious friends. The 'old doctor' had effected wonders, and he received, as he should, great credit for it.

To some, the means employed may not seem adequate to the end obtained, especially when they are informed, that the next day I gave a patient, in which there was no obstruction in the intestinal canal, nearly half a pint of *that same aromatic syrup of rhubarb*, without producing even a laxative effect.

On the 11th of May, 1843, I visited E. B. J., a plasterer, of rather a delicate constitution, about 29 years old. He was taken sick on the 9th, with a sharp pain in the left side, extending up into the left shoulder joint. He informed me that he had never had rheumatism, but that about 8 years ago he had fever with cough and acute pain in the left side. The pain in the present instance was entirely and permanently removed by a single bleeding, and a few small portions of tartar emetic, nor did he after this, throughout the whole period of his sickness have either cough, or hurried, or difficult respiration.

Notwithstanding the removal of the pain, the fever increased daily, the pulse becoming hard and corded, and as there were now no symptoms present calculated to direct the attention to any particular organ—(the stethescope giving no indications of disease of the lungs or pleura)—I concluded to examine the state of the heart. Synchronous with the first sound, and continuous with its whole duration, was heard a little below the left nipple, midway between it and the centre of the sternum, a moderately low and rather harsh bellows murmur. More to the left, say half an inch to the right of the nipple, and an inch and a quarter below it, and from this point to the nipple, was heard a low creaking sound synchronous with the second sound of the heart, and continued into the commencement of the first sound—that is, it was heard principally during the diastole—but also slightly during the commencement of the systole of the heart. The sound seemed superficial, was loud and distinct, and very closely resembled the creaking of the sole of a new boot. During the progress of the case the sound was frequently modified, at times even temporarily removed by treatment. For instance, for some hours after a copious bloodletting, by which the force of the pulse was considerably reduced, the sound entirely subsided. As the pulse gathered strength, the sound gradually returned, at first only to be heard during the period of rest after exhaustion, and to disappear when the chest was fully expanded. Then, when the heart again acted strongly, to be heard plainly, both when the chest was fully expanded, and when it was collapsed.

I found the diagnosis of the endo-pericarditis, the sound last described, not only deciding my opinion as to the affection of the *pericardium*, but also influencing me considerably in the treatment. This was decidedly antiphlogistic, and in no case have I ever seen the propriety of antiphlogistic measures justified by so thick a buffy coat, or so firm a crassamentum.

The case terminated fatally about the commencement of the 9th week; the pulse continuing hard and jerking almost to the last hour.

In one respect, the sound alluded to differed from the attrition murmur of pericarditis, as described by Hope. It will be remembered that in regard to the first sound of the heart, it was only at its commencement that it (the morbid sound) was heard. He says, "the murmur is almost always double, accompanying the two sounds of the heart, in correspondence with the movements of the organs backwards and forwards within the pericardium. I have, however, occasionally found it *stronger with the first sound, and once or twice I have heard it accompany that sound exclusively.* This might be anticipated, in consequence of the superior force of the systolic movement." In my case it was with the diastolic movement that the sound was principally heard; nevertheless

I could think of nothing capable of producing such a sound, except the effusion of tolerably firm coagulable lymph within the pericardium.

In a post-mortem examination, a few very soft and delicate masses of coagulable lymph were found on the surface of the heart, but entirely inadequate to the production of so harsh and loud a sound. In the right auricle and ventricle was a very firm fibrinous concretion, matted in the tricuspid valve and chorda tendineæ, and at points connected intimately with the smoother parts of the internal surface of the heart. So perfect was its organization that it required some little examination to distinguish the morbid formation from the natural structure of the heart. It contained no red globules, and had an outer coat, which could be peeled off in delicate shreds of an equable thickness, having much the appearance of an organized serous covering. A couple of small unadherent coagula were found in the left ventricle. The tricuspid, the aortic, and the bicuspid valves were slightly thickened and much redder than natural. This was more particularly the case with the latter, which was likewise studded thickly with semi-cartilaginous deposits, from the size of a poppy seed, to that of a flaxseed. About an inch within the aorta were several small white patches, rough, as if from absorption of the lining membrane. At the base of each of the aortic valves was a small ossific deposit.

As yet I had discovered nothing capable of satisfactorily explaining the creaking sound. Placing the heart back *in situ*, I proceeded to an examination of the lungs. The right was entirely healthy; the left contained a very few scattered tubercles, and was throughout firmly adherent to the parietes, to the diaphragm, and to the pericardium, the result no doubt of a former attack. In its thin edge, which was adherent to the pericardium, and which overlapped the left ventricle, a small portion of the pulmonary tissue had undergone a kind of semi-cartilaginous degeneration. This, by sudden pressure and relaxation between the finger and thumb, produced a sound exactly like that heard during the life of the patient.

The part of the sound produced by the pressure corresponding with that which accompanied the second sound of the heart, and that produced by the relaxation, corresponding as well with that part of it which was synchronous with the commencement of the first sound.

In this manner, so precisely could I imitate the morbid sound heard during the life of the patient, as to leave no doubt on my mind, that it was the motions of the heart against this substance which produced it.

Although my diagnosis happened in the main to be correct, yet the very symptom which went further than any other to determine that diagnosis, proved to have no connection whatever with the disease.

*Case of Amaurosis occurring during an attack of Congestive Fever.*

February 26th, 1844—Called to see Mrs. H., a lady about 24 years old, who has generally, until within the last few months, enjoyed good health. She is pregnant, for the first time, and she supposes in about her eighth month. During the whole period of her pregnancy she has suffered more from nausea and vomiting, than females in her condition generally do.

On the morning of the 24th, she had some slight chilly sensations, followed by high fever, and increased nausea and vomiting, the matter ejected being of a rich dark brown, nearly black color, and viscid, much resembling the appearance produced by the partial suspension and partial solution of pulverized extract of liquorice in a mucilage of slippery elm. The quantity thrown up was considerable, and recurred about every half hour.

On the morning of the 25th, the chilly sensations again returned, and this morning also, but without being preceded by any thing like a remission in the fever, or any abatement in the vomiting. Since the chilly sensations this morning, the voming has increased, the intervals between each spell being only about fifteen minutes. For a sensation of fullness about the head, she was bled yesterday to ℥xii, with relief to that symptom.

Her situation at the time of my first visit (1 o'clock, P.M., 26th) was as follows:—Pulse 145, small and weak; respiration natural; tongue moist, rough and white; countenance not expressive of much suffering or anxiety; skin cool, more especially that of the extremities, and of a livid ashy color. There is *less nausea* than usually accompanies so much vomiting. Each effort to vomit is preceded by hiccough and a burning sensation in the stomach, and followed by a burning sensation in the œsophagus and mouth. The matter thrown up is, she says, sometimes insipid, sometimes "bitterish sour" to the taste; no evacution from bowels in 48 hours. She has taken nothing but a few small portions of morphine and tinct. opii, all of which have been soon thrown up. She has suffered some from pains in her limbs, but much more from pain in her back.

I applied sinapisms to her extremities, abdomen and spine, and ordered an enema, made active by a small handful of table-salt, and immediately after a spell of vomiting, administered a third of a grain of morphine, so that there might be time for it to produce some effect on the stomach before the probable occurrence of the next spell. In two hours she had had no return of vomiting, pain in the back much relieved, and she had had several small evacutions of hardened feces. ℞ quin. sulph. ℈iiss., ext. glycyrrh. grs. xviii. M. fiat massa in pilulas xii. divid.; two of them to be taken every third hour. Small quantities of brandy toddy occasionally, and a little lime water in the water she drinks.

Late in the evening Dr. Ames saw her in consultation with me. There had been no return of the vomiting, and the burning sensation in the stomach was quite relieved; pulse 128. The same treatment to be continued, (the quinine only every fourth hour), the enema to be repeated, and should the vomiting return, the morphine also.

27th.—Dr. Jones, the family physician, who has been absent, has returned, and joins us in the management of the case. She has vomited twice during the night, the matter, however, being less viscid, and the dark flocculi smaller in quantity. Several thin, yellow evacutions; feels quite faint; pulse 140; respiration 12; took two doses of morphine, a third of a grain each; continue the quinine pills and repeat the morphine; should she vomit again, continue the toddy.

Evening.—No vomiting, no evacuation. In the fore part of the day she had a great desire to be fanned, and to have her face and extremities bathed in cold water. Pulse fallen to 136; respiration 16; extremities warm; continue the same treatment.

28th.—She has not vomited, but had early last night considerable nausea, to relieve which she took a third of a grain of morphine; after this she slept about half an hour and awoke completely blind, not even having any perception of light, when the glare of a lighted candle was thrown on her eyes, and within a few inches of them—this state continues.

She has the inexpressive stare of amaurosis, and is in a great measure unable to control the motions of the eyes. The pupils dilate and contract irregularly, the dilatation being greatest when she is requested to make an effort to see any object. Pulse 124; respiration 16; temperature of the skin nearly natural. Continue quinine, grs. iv., every fourth hour.

29th.—Pulse 100, full and soft; no thirst; some appetite; pupils very much dilated and not at all affected by any degree of light. She is left to-day under the care of Dr. Jones, the treatment agreed on being an occasional mercurial laxative, and free cupping from the back of the neck.

Although we had considerable anxiety on account of the state of her eyes, we had strong hopes that her vision would be promptly restored after parturition, even if no improvement took place before that time.

On the evening of the 29th, labour pains came on, which Dr. Jones, who was present, thinking premature, attempted to allay with morphine. After the first portion of morphine her pupils became very much contracted.

The morphine moderated, but did not entirely subdue the pains, and on the evening of March 1st, as they again became more severe, it was deemed unnecessary to interfere further. Accordingly about 10 o'clock at night she was delivered of a dead child, which appeared to have completed the ninth month.

No immediate improvement in vision took place after delivery.

March 3d.—Dr. Sims and myself visited her in consultation with Dr. Jones. The cupping has not been carried out effectually on account of her repugnance to the operation. Last night a blister was applied to the back of her neck. The pupils have again become dilated, and are slightly influenced by light. She has at times a slight perception of light when the luminous object is situated laterally, and this slight perception, is rendered more distinct after she has sat up in bed sometime. Dr. Sims and myself thought at one time that there was a slight degree of haziness in the interior of the eye, but concurred in the opinion of Dr. Jones afterwards that it was imaginary. Three images of the candle visible in the catoptric examination of the eye. Pulse 80; appetite tolerable; some thirst, and tongue slightly furred. ℞ calomel grs. x., compound extract of colocynth grs. v, immediately. After its operation, a moderately loose state of the bowels to be kept up by small doses of blue mass, till ptyalism is induced. Blisters on neck to be kept running; cups occasionally to the temples; moderately nourishing diet.

9th.—Mouth a little sore; vision very slightly improved; complained much yesterday of pains darting across from one eye to the other; continue same treatment.

17th.—Dr. Ames again visited her with us; mouth less sore; (the mercurial having been omitted). She can now tell when any one passes between her and a bright light. Pupils less dilated, but still preternaturally so; her color and strength have improved but slowly; she is able, however, to walk across the room unsupported; tongue rough and coated; appetite pretty good; bowels costive; milk still secreted by the mammæ. ℞ ferri. carb. precip. grs. x. three times a day; blue mass grs. v. twice a day, and aloes in sufficient quantity to keep her bowels moderately open should the blue mass fail to do so; a more nutritious diet; tartar emetic ointment to the back of her neck, and the endermic application of strychnine to the temples.

28th.—There has been a slight improvement in her vision; catamenia re-established; continue the same treatment; substituting a weak solution of strychnine to the eyes for its application to the blistered surface on the temples.

May 12th.—All treatment has been suspended for four or five weeks. Since the suspension her strength and general health have improved considerably, but her vision none. Up to the time of the suspension of treatment, her vision had gradually improved, and that improvement still remains. She can see, but rather imperfectly, to read small print. Eyes easily fatigued, and when so, there is considerable twitching of the lids, and unsteadiness of the balls. Pupils still preternaturally dilated, and not regularly influenced by light. Ordered to resume the mercurials, blisters to neck and temples, and also the strychnine.

16th.—Her husband tells me that there has been a slight improvement. He thinks that after the drawing of a blister on one temple, the corresponding eye seems smaller, or less prominent.

As she is about to visit a relation at the North, where she will remain some months, she ceases to be under my care.

As to whether the quinine which she took, had any agency in producing the blindness, I will not pretend to decide. It will be seen that in about 30 hours preceding the blindness she took nearly a drachm. It affected the auditory nerves rather less than the same quantity usually does. Although I have for a number of years used the remedy extensively in practice, it is the first time that any affection of the organs of vision has supervened in my hands during its administration. Two or three years ago a case of amaurosis, under nearly similar circumstances, occurred in the practice of an acquaintance of mine. The subject of it was a plasterer, about 35 years old. He had a severe attack of congestive fever, for which he was treated principally with quinine, in large doses. Before convalescence commenced, he suddedly became entirely blind, and remained so for some weeks after his recovery from the fever. After this his vision gradually improved, so that in a few months he was able to superintend business, but remains still very imperfect.

The quantity of quinine administered in the case, of which I have given the details, was less than I have prescribed in hundreds of other cases, without producing any analogous effects, but as the remedy has recently come into such general use in this part of the country, and I believe in many other parts of the south and south-west, as to have almost usurped the place which calomel occupied a few years since; mention of its possible agency in producing the effects alluded to, may not be deemed superfluous. For it is a fact, which many must have observed, that where any particular remedy is fast advancing in public estimation, its reputation is apt to exceed the proper limits, and consequently lead to its profuse, indiscriminate, and incautious administration; and the more valuable the remedy, to a greater extent is this the case. Something like this, no doubt, is, in a great measure, the cause of the rapid fall of calomel from that high place which but recently it held, both with the profession, and the public generally.

July 22, 1844.

